# Supplementary material for: Association between occupational exposures and chronic low back pain: Protocol for a systematic review and meta-analysis
Source: PLoS One. 2023 May 5;18(5):e0285327. doi: 10.1371/journal.pone.0285327 (PMC10162539; doi:10.1371/journal.pone.0285327)
Supplement: S2 Appendix — (PDF) [file pone.0285327.s002.pdf]

Search strategy

Block 1

Mesh	Free text search in title and abstract
”Occupations”[Mesh]<br>”Occupational Health”[Mesh]<br>”Occupational Diseases”[Mesh]<br>”Occupational Exposure”[Mesh]<br>”Occupational Groups”[Mesh]<br>”Work”[Mesh]<br>”Workplace”[Mesh]<br>”Workload”[Mesh]<br>”Women, Working”[Mesh]<br>”Employment”[Mesh]	occupation*<br>employ*<br>job*<br>working condition*<br>work-related<br>work-load*<br>work-place*<br>work environment*

Block 2

Mesh	Free text search in title and abstract
**Mechanical stress**<br>”Stress, Mechanical”[Mesh]	
**Organisational and psychosocial factors:**<br>"Stress, Psychological”[Mesh]	
**Development of force**:<br>”Lifting”[Mesh]<br>”Weight-Bearing”[Mesh] ”Biomechanics”[Mesh]<br>”Moving and Lifting Patients”[Mesh]<br>”Physical Exertion”[Mesh]	**Development of force**:<br>lift*, carry*, hold*, pull*, drag*, push*, manual handling, force*, biomechanic*, physical demand*, physically demand*.
**Working posture:**<br>”Torsion, Mechanical”[Mesh]<br>”Postural Balance”[Mesh]<br>”Walking”[Mesh]	**Working posture:**<br>flexion*, extension*, turning*, sitting*, kneeling*, twisting*, bending, sedentary, walking*, reaching, squatting, standing, postural balance, static AND posture, awkward AND posture.
**Working movement:**<br>”Recovery of Function”[Mesh] ”Relaxation”[Mesh]	**Working movement:**<br>repetitive movement*, monotonous work, dynamic AND posture, relaxation, recovery of function, static work, dynamic load.
**Influence and demand:**	**Influence and demand:**<br>decision latitude, work demand*, job demand*, high demand*, low control, work control, job control, work influence*, demand resource*, lack of control, job strain, work strain.
**Effort and reward:**	**Effort and reward:**<br>effort reward*, time pressure*, work overload*, recuperation*, recovery.
**Social support and relations in the workplace:**<br>”Social Support”[Mesh]<br>”Employee Performance "Appraisal”[Mesh]<br>”Organizational Culture”[Mesh]<br>"Justice/psychology”[Mesh]<br>”Communication/psychology”[Mesh]<br>”Interpersonal Relations”[Mesh]	**Social support and relations in the workplace:** social support, support system*, social network*, emotional support, justice*, injustice*, interaction*, interpersonal relation*.
**Job satisfaction:**<br>”Job Satisfaction”[Mesh]<br>”Employee Grievances”[Mesh]	**Job satisfaction:**<br>Boredom, job satisfaction, work satisfaction, coping, work ability.

**Education and learning:**<br>"Staff Development"[Mesh]	**Education and learning:**<br>skill discretion*, staff development.
**Conflict, violence or harassment:**<br>"Bullying"[Mesh]<br>"Prejudice"[Mesh]<br>"Social Discrimination"[Mesh]	**Conflict, violence or harassment:**<br>harass*, workplace conflict*, workplace violen*, silent workplace*, victimization*, bullying, role ambiguity, role conflict*, work role*, discrimination.
**Working time:**<br>"Work Schedule Tolerance"[Mesh]	**Working time:**<br>working hour*, working time, shift work*, work shift*, day-time, night-time, temporary work, full-time, part-time, flexible work*, lean production.
**Job insecurity:**<br>"Personnel Downsizing"[Mesh]	**Job insecurity:**<br>organizational change, job security, job insecurity.
**Chemical and biological substances:**<br>"Air Pollution"[Mesh]<br>"Air Pollutants"[Mesh]<br>"Solvents"[Mesh]<br>"Fluids and Secretions"[Mesh]	**Chemical and biological substances:**<br>pollut*, indoor air*, airborne, passive smok*, solvent*, smok* AND pollute*.
**Contact with chemicals:**<br>"Toxic Actions"[Mesh]	**Contact with chemicals:**<br>hazardous chemical*, hazardous material*, hazardous substance*, toxic action*, pesticide*, poison*.
**Noise:**<br>"Noise"[Mesh]	**Noise:**<br>noise*
**Vibrations:**<br>"Vibration"[Mesh]<br>"Automobile Driving"[Mesh]<br>"Motor Vehicles"[Mesh]	**Vibrations:**<br>vibrat*, driving, vehicle*, truck*, lorry/lorries, automobile*, car/cars, buses, hand tool*, hand-held tool*, power tool*.
**Radiation:**<br>"Radiation"[Mesh]<br>"Air Pollution, Radioactive"[Mesh]	**Radiation:**<br>radiation*
**Temperature:**<br>"Hot Temperature"[Mesh]<br>"Cold Temperature"[Mesh]<br>"Climate"[Mesh]	**Temperature:**<br>climate*, cold temperature*, hot temperature*.
**Infected materials:**<br>"Communicable Diseases"[Mesh]	**Infected materials:**<br>contagious* communicable disease*

Block 3

Mesh	Free text search in title and abstract
**Back problems – anatomy and disease:**<br>"Back" [Mesh]<br>"Spine" [Mesh]<br>"Pelvis" [Mesh]<br>"Pain" [Mesh]<br>"Pain Measurement" [Mesh]<br>"Cumulative Trauma Disorders" [Mesh] "Musculoskeletal Diseases" [Mesh]<br>"Back Pain" [Mesh]<br>"Back Injuries" [Mesh]<br>"Spinal Diseases" [Mesh<br>"Pelvic Pain" [Mesh]<br>"Sciatica" [Mesh]	**Back problems – anatomy and disease:**<br>back, spine*, spinal*, trunk*, lumbar*, pelvis*, sacrum, lumbo-sacral*, lumbosacral*, intervertebral disk*, intervertebral disc*, thoracic vertebrae, thoracic vertebra.<br><br>pain, ache*, musculoskeletal disease*, musculoskeletal disorder*, cumulative trauma disorder*, nerve entrapment.<br><br>back pain, backache*, back injur*, spinal disease*, spine disease*, spinal injur* OR spine injur*, intervertebral disk degeneration, intervertebral disc degeneration, spinal osteochondros*, spine osteochondros*, Scheuermann*, spinal stenos*, spondylitis, spondylarthritis, spondylosis, lumbago, sciatica, pelvic pain.

Block 4

Limits: English, Danish, Norwegian, Swedish.
Publication Date: from 2014/01/01 to 2021/09/20.

Search string

((("Back"[MeSH Terms] OR "spine"[MeSH Terms] OR "Pelvis"[MeSH Terms] OR ("Back"[Title/Abstract] OR "spine*"[Title/Abstract] OR "spinal*"[Title/Abstract] OR "trunk*"[Title/Abstract] OR "lumbar*"[Title/Abstract] OR "pelvis*"[Title/Abstract] OR "sacrum"[Title/Abstract] OR "lumbo sacral*"[Title/Abstract] OR "lumbosacral*"[Title/Abstract] OR "intervertebral disk*"[Title/Abstract] OR "intervertebral disc*"[Title/Abstract] OR "thoracic vertebrae"[Title/Abstract] OR "thoracic vertebra"[Title/Abstract])) AND ("Pain"[MeSH Terms] OR "Pain Measurement"[MeSH Terms] OR "Cumulative Trauma Disorders"[MeSH Terms] OR "Musculoskeletal Diseases"[MeSH Terms] OR ("Pain"[Title/Abstract] OR "ache*"[Title/Abstract] OR "musculoskeletal disease*"[Title/Abstract] OR "musculoskeletal disorder*"[Title/Abstract] OR "cumulative trauma disorder*"[Title/Abstract] OR "nerve entrapment"[Title/Abstract]))) OR ("Back Pain"[MeSH Terms] OR "Back Injuries"[MeSH Terms] OR "Spinal Diseases"[MeSH Terms] OR "Pelvic Pain"[MeSH Terms] OR "Sciatica"[MeSH Terms] OR ("Back Pain"[Title/Abstract] OR "backache*"[Title/Abstract] OR "back injur*"[Title/Abstract] OR "spinal disease*"[Title/Abstract] OR "spine disease*"[Title/Abstract] OR "spinal injur*"[Title/Abstract] OR "spine injur*"[Title/Abstract] OR "intervertebral disk degeneration"[Title/Abstract] OR "intervertebral disc degeneration"[Title/Abstract] OR "spinal osteochondros*"[Title/Abstract] OR "spine osteochondros*"[Title/Abstract] OR "scheuermann*"[Title/Abstract] OR "spinal stenos*"[Title/Abstract] OR "spondylitis"[Title/Abstract] OR "spondylarthritis"[Title/Abstract] OR "spondylosis"[Title/Abstract] OR "lumbago"[Title/Abstract] OR "Sciatica"[Title/Abstract] OR "Pelvic Pain"[Title/Abstract]))) AND ("Work"[MeSH Terms] OR "Workload"[MeSH Terms] OR "Workplace"[MeSH Terms] OR "Occupations"[MeSH Terms] OR "Occupational Health"[MeSH Terms] OR "Occupational Diseases"[MeSH Terms] OR "Occupational Groups"[MeSH Terms] OR "Occupational Exposure"[MeSH Terms] OR "women, working"[MeSH Terms] OR "Employment"[MeSH Terms:noexp] OR (("work-related"[Title/Abstract] OR "work load*"[Title/Abstract] OR "workload*"[Title/Abstract] OR "workplace*"[Title/Abstract] OR "workplace*"[Title/Abstract] OR "work environment*"[Title/Abstract] OR "working condition*"[Title/Abstract] OR "occupation*"[Title/Abstract] OR "job"[Title/Abstract] OR "employ*"[Title/Abstract]) NOT ("medline"[Filter] OR "oldmedline"[Filter]))) AND ("stress, mechanical"[MeSH Terms] OR "Lifting"[MeSH Terms] OR "Moving and Lifting Patients"[MeSH Terms] OR "Weight-Bearing"[MeSH Terms] OR "Physical Exertion"[MeSH Terms] OR "torsion, mechanical"[MeSH Terms] OR "Postural Balance"[MeSH Terms] OR "Walking"[MeSH Terms] OR "recovery of function"[MeSH Terms] OR "Relaxation"[MeSH Terms] OR ("static"[Title/Abstract] AND ("postural"[All Fields] OR "posturally"[All Fields] OR "posture"[MeSH Terms] OR "posture"[All Fields] OR "postures"[All Fields] OR "postured"[All Fields] OR "posturing"[All Fields])) OR ("awkward"[Title/Abstract] AND ("postural"[All Fields] OR "posturally"[All Fields] OR "posture"[MeSH Terms] OR "posture"[All Fields] OR "postures"[All Fields] OR "postured"[All Fields] OR "posturing"[All Fields])) OR ("dynamic"[Title/Abstract] AND ("postural"[All Fields] OR "posturally"[All Fields] OR "posture"[MeSH Terms] OR "posture"[All Fields] OR "postures"[All Fields] OR "postured"[All Fields] OR "posturing"[All Fields])) OR "static work"[Title/Abstract] OR "dynamic load*"[Title/Abstract] OR "lift*"[Title/Abstract] OR "carry*"[Title/Abstract] OR "hold*"[Title/Abstract] OR "pull*"[Title/Abstract] OR "drag*"[Title/Abstract] OR "push*"[Title/Abstract] OR "manual handling"[Title/Abstract] OR "force*"[Title/Abstract] OR "biomechanic*"[Title/Abstract] OR "walking*"[Title/Abstract] OR "Postural Balance"[Title/Abstract] OR "flexion*"[Title/Abstract] OR "extension*"[Title/Abstract] OR "turning"[Title/Abstract] OR "sitting"[Title/Abstract] OR "kneeling"[Title/Abstract] OR "squatting"[Title/Abstract] OR "twisting"[Title/Abstract] OR "bending"[Title/Abstract] OR "reaching"[Title/Abstract] OR "standing"[Title/Abstract] OR "sedentary"[Title/Abstract] OR "repetitive movement*"[Title/Abstract] OR "monotonous work"[Title/Abstract] OR "Relaxation"[Title/Abstract] OR "recovery of function"[Title/Abstract] OR "physical demand*"[Title/Abstract] OR "physically demand*"[Title/Abstract] OR ("stress, psychological"[MeSH Terms] OR "Social Support"[MeSH Terms] OR "Job Satisfaction"[MeSH Terms] OR "Work Schedule Tolerance"[MeSH Terms] OR "Employee Performance Appraisal"[MeSH Terms] OR "Employee Grievances"[MeSH Terms] OR "social justice/psychology"[MeSH Terms] OR "Personnel Downsizing"[MeSH Terms] OR "Staff Development"[MeSH Terms] OR "Organizational Culture"[MeSH Terms] OR "Bullying"[MeSH Terms] OR "Prejudice"[MeSH Terms] OR "Social Discrimination"[MeSH Terms] OR "Interpersonal Relations"[MeSH Terms] OR "communication/psychology"[MeSH Terms] OR "psychosocial"[Title/Abstract] OR "job strain"[Title/Abstract] OR "work strain"[Title/Abstract] OR "work demand*"[Title/Abstract] OR "job demand*"[Title/Abstract] OR "high demand*"[Title/Abstract] OR "low control"[Title/Abstract] OR "lack of control"[Title/Abstract] OR "work control"[Title/Abstract] OR "job control"[Title/Abstract] OR "decision latitude"[Title/Abstract] OR "work influence*"[Title/Abstract] OR "demand resource*"[Title/Abstract] OR "effort reward*"[Title/Abstract] OR "time pressure*"[Title/Abstract] OR "recuperation*"[Title/Abstract] OR "work overload*"[Title/Abstract] OR "work over load*"[Title/Abstract] OR "recovery"[Title/Abstract] OR "coping"[Title/Abstract] OR "work ability"[Title/Abstract] OR "Social Support"[Title/Abstract] OR "support system*"[Title/Abstract] OR "social network*"[Title/Abstract] OR "emotional support"[Title/Abstract] OR "interpersonal relation*"[Title/Abstract] OR "interaction*"[Title/Abstract] OR

"justice*"[Title/Abstract] OR "injustice*"[Title/Abstract] OR "Job Satisfaction"[Title/Abstract] OR "work satisfaction"[Title/Abstract] OR "boredom"[Title/Abstract] OR "skill discretion*"[Title/Abstract] OR "Staff Development"[Title/Abstract] OR "discrimination"[Title/Abstract] OR "harass*"[Title/Abstract] OR (("workplace"[MeSH Terms] OR "workplace"[All Fields] OR ("work"[All Fields] AND "place"[All Fields]) OR "work-place"[All Fields]) AND "conflict*"[Title/Abstract]) OR "workplace violen*"[Title/Abstract] OR "work place violen*"[Title/Abstract] OR "Bullying"[Title/Abstract] OR "victimization*"[Title/Abstract] OR (("silent"[All Fields] OR "silently"[All Fields] OR "silents"[All Fields]) AND "workplace*"[Title/Abstract]) OR "role ambiguity"[Title/Abstract] OR "roleconflict*"[Title/Abstract] OR "work role*"[Title/Abstract] OR "working hour*"[Title/Abstract] OR "working time"[Title/Abstract] OR "daytime"[Title/Abstract] OR "night-time"[Title/Abstract] OR "shift work*"[Title/Abstract] OR "work shift*"[Title/Abstract] OR "temporary work*"[Title/Abstract] OR "full-time"[Title/Abstract] OR "part-time"[Title/Abstract] OR "flexible work*"[Title/Abstract] OR "organizational change"[Title/Abstract] OR "organisational change"[Title/Abstract] OR "lean production"[Title/Abstract] OR "job security"[Title/Abstract] OR "job insecurity"[Title/Abstract]) OR ("Air Pollutants"[MeSH Terms] OR "Air Pollution"[MeSH Terms] OR "Fluids and Secretions"[MeSH Terms] OR "Toxic Actions"[MeSH Terms] OR "Solvents"[MeSH Terms] OR "pollut*"[Title/Abstract] OR "indoor air*"[Title/Abstract] OR "airborne"[Title/Abstract] OR ("smok*"[Title/Abstract] AND "pollut*"[Title/Abstract]) OR "passive smok*"[Title/Abstract] OR "hazardous chemical*"[Title/Abstract] OR "hazardous material*"[Title/Abstract] OR "hazardous substance*"[Title/Abstract] OR "toxic action*"[Title/Abstract] OR "pesticide*"[Title/Abstract] OR "poison*"[Title/Abstract] OR "solvent*"[Title/Abstract]) OR ("Radiation"[MeSH Terms] OR "air pollution, radioactive"[MeSH Terms] OR "Hot Temperature"[MeSH Terms] OR "Cold Temperature"[MeSH Terms] OR "Climate"[MeSH Terms] OR "radiation*"[Title/Abstract] OR "climate*"[Title/Abstract] OR "cold temperature*"[Title/Abstract] OR "hot temperature*"[Title/Abstract]) OR ("Communicable Diseases"[MeSH Terms] OR "communicable disease*"[Title/Abstract] OR "contagious*"[Title/Abstract]) OR ("Noise"[MeSH Terms] OR "noise*"[Title/Abstract]) OR ("Vibration"[MeSH Terms] OR "Motor Vehicles"[MeSH Terms] OR "Automobile Driving"[MeSH Terms] OR "driving"[Title/Abstract] OR "automobile*"[Title/Abstract] OR "car"[Title/Abstract] OR "cars"[Title/Abstract] OR "vibrat*"[Title/Abstract] OR "vehicle*"[Title/Abstract] OR "truck*"[Title/Abstract] OR "lorry"[Title/Abstract] OR "lorries"[Title/Abstract] OR "buses"[Title/Abstract] OR "hand held tool*"[Title/Abstract] OR "hand tool*"[Title/Abstract] OR "power tool*"[Title/Abstract])) AND ("english"[Language] OR "danish"[Language] OR "norwegian"[Language] OR "swedish"[Language])
