# Supplementary material for: Association between occupational exposures and chronic low back pain: Protocol for a systematic review and meta-analysis
Source: PLoS One. 2023 May 5;18(5):e0285327. doi: 10.1371/journal.pone.0285327 (PMC10162539; doi:10.1371/journal.pone.0285327)
Supplement: S3 Appendix — (PDF) [file pone.0285327.s003.pdf]

PECOS

Population
Inclusion
Adults in or above working age.
Exclusion
Adults never in work or left the labour market at the beginning of the study.
Students.

Exposure
Inclusion
Occupational mechanical exposures (e.g. working posture, lift, and development of force, whole body vibration and vibrations from handheld tools, person lifting and moving)
Occupational psychosocial exposures (e.g. job demand, workload, harassment, conflict, social relation, and support).
Quantified exposure measure through self-report, interview, observation, technical measure or job-exposure-matrices (JEM).
Exclusion
Exposures related to chemical or biological substances, radiation, heat, cold or accidents/injuries.
Exposures based solely on job titles or non-related occupational exposures.

Comparison
Studies will only be included if the effect size on the association between occupational exposures and chronic low back pain is expressed in an appropriate risk estimate or possible to calculate.

Outcome
Inclusion
Chronic low back pain including lumbago/sciatica, lumbar herniated disc/protrusion and lumbosacral degenerative changes with or without radiculopathy.
Low back pain lasting ≥3 months.
Outcome measured with self-report, interview, clinical diagnosis, surgery or another measure (e.g. x-ray, insurance).
Exclusion
Occupational injuries based on accidents.
Inherent pain not caused by occupational mechanical exposures.
Pain caused by other diseases or conditions than those described in the inclusion, such as cancer, fracture or inflammation related to the lower back.
Studies reporting a proxy to chronic low back pain (e.g. sickness absenteeism).
Studies not reporting chronic low back pain.

Study design
Inclusion
Randomised control trial studies.
Cohort studies.
Case-control studies.
Original study in full text and peer-reviewed.
In English, Danish, Swedish or Norwegian.

Published from January 2014 and forward.
Exclusion
Cross-sectional studies.
Systematic reviews.
In vitro studies.
Studies solely based on health economics.
Studies not addressing any risk factors related to work (e.g. treatment, prognostic or rehabilitation).
Studies with less than 30 participants.
Animal trials.
Conference notes, books, letters to editor, editorial pages, protocols, reports and abstracts.
Studies in other languages than those described in the inclusion.
