# Supplementary material for: Association between occupational exposures and chronic low back pain: Protocol for a systematic review and meta-analysis
Source: PLoS One. 2023 May 5;18(5):e0285327. doi: 10.1371/journal.pone.0285327 (PMC10162539; doi:10.1371/journal.pone.0285327)
Supplement: S4 Appendix — (PDF) [file pone.0285327.s004.pdf]

Critical appraisal tool

Case-Control Study	Yes	No	Unclear
**Major Risk 1 – study design and selection**			
**Were the cases recruited in an acceptable way?**<br><br>Consider the following:<br>■ Are the cases representative of a population, clearly defined and differentiated from controls?<br>■ Was there an established reliable system for selecting all the cases?<br>■ Were inclusion and exclusion criteria explicit and applied similarly to all eligible cases?			
**Were the controls selected in an acceptable way?**<br><br>Consider the following:<br>■ Are the controls representative of a population and clearly defined?<br>■ Are the same inclusion and exclusion criteria for cases used to select controls (equally applied) and matched appropriately?<br>■ Is it clearly established that controls are non-cases?			
**Is the participation rate satisfactory?**<br><br>Consider the following:<br>■ Are there large differences between the two groups?<br>■ Is the participation rate low?			
**Major risk 2 - Exposure**			
**Was the exposure accurately measured to minimise bias?**<br><br>Consider the following:<br>■ Is the exposure clearly defined?<br>■ Do measurements truly reflect what it is supposed to measure (have they been validated?).			

■ Is the method of assessment reliable?			
**Major risk 3 – Outcome**			
**Was the outcome accurately measured to minimise bias?**<br><br>Consider the following:<br>■ Is the outcome clearly defined?<br>■ Do measurements truly reflect what it is supposed to measure (have they been validated?).<br>■ Is the method of assessment reliable?			
**Major risk 4 – Non-participants**			
**Is comparison made between participants and non-participants?**<br><br>Consider the following:<br>■ Is similarities or differences established?			
**Major risk 5 – Analysis method**			
**Was the analysis method adequate?**<br><br>Consider the following:<br>■ Are the main potential confounders identified and taken into account in the analysis?<br>■ Were adequate statistical models used to reduce bias?			
**Minor risk 1 – Funding**			
**Was the source of funding provided?**<br><br>Consider the following:<br>■ Was the study affected by sponsors?<br>■ Did sponsoring organization participate in the analysis?			
**Minor risk 2 – Chronology**			

**Could chronology be established?**<br><br>Consider the following:<br>■ Was the timeframe sufficient to see an association between the exposure and outcome?			
**Minor risk 3 – Conflict of interest**			
**Was the study without any conflict of interest?**<br><br>Consider the following:<br>■ Was the study affected by the authors affiliations or interests?			

Cohort Study	Yes	No	Unclear
**Major Risk 1 – study design and selection**			
**Was the cohort recruited in an acceptable way?**<br><br>Consider the following:<br>■ Is it representative of a defined population and clearly specified?<br>■ Are groups comparable in all respects other than the factor under investigation?<br>■ Was everybody included who should have been?			
**Was the follow-up of subjects acceptable?**<br><br>Consider the following:<br>■ Conventionally, a 20% drop out rate is acceptable, but observational studies conducted over longer periods, a higher drop-out rate is to be expected.<br>■ Were losses to follow-up taken into account in the analysis (sensitivity analysis, described etc.)?			
**Major risk 2 - Exposure**			
**Was the exposure accurately measured to minimise bias?**<br><br>Consider the following:			

■ Is the exposure clearly defined?<br>■ Do measurements truly reflect what it is supposed to measure (have they been validated?).<br>■ Is the method of assessment reliable?<br>■ Were all the subjects classified into exposure groups using the same procedure?			
**Major risk 3 – Outcome**			
**Was the outcome accurately measured to minimise bias?**<br><br>Consider the following:<br>■ Is the outcome clearly defined?<br>■ Do measurements truly reflect what it is supposed to measure (have they been validated?).<br>■ Is the method of assessment reliable?<br>■ Were the measurement methods similar in the different groups?<br>■ If blinding is not possible, is there some recognition that knowledge of exposure status could influence the assessment of the outcome?			
**Major risk 4 – Enrolment**			
**Was the outcome taken into account at enrolment?**<br><br>Consider the following:<br>■ Some participants might have the outcome at the time of enrolment. Is it assessed at baseline in the analysis?			
**Major risk 5 – Analysis method**			
**Was the analysis method adequate?**<br><br>Consider the following:<br>■ Are the main potential confounders identified and taken into account in the analysis?<br>■ Were adequate statistical models used to reduce bias?			
**Minor risk 1 – Funding**			
**Was the source of funding provided?**			

Consider the following:<br>■ Was the study affected by sponsors?<br>■ Did sponsoring organization participate in the analysis?			
**Minor risk 2 – Chronology**			
**Could chronology be established?**<br><br>Consider the following:<br>■ Was the timeframe sufficient to see an association between the exposure and outcome?<br>■ Was the follow-up long enough for the outcome to occur?			
**Minor risk 3 – Conflict of interest**			
**Was the study without any conflict of interest?**<br><br>Consider the following:<br>■ Was the study affected by the authors affiliations or interests?			
