# Supplementary material for: Association between occupational exposures and chronic low back pain: Protocol for a systematic review and meta-analysis
Source: PLoS One. 2023 May 5;18(5):e0285327. doi: 10.1371/journal.pone.0285327 (PMC10162539; doi:10.1371/journal.pone.0285327)
Supplement: Attachment — Submitted filename: Response to Reviewers.docx [file pone.0285327.s005.docx]

Date 08.09.2022, PLOS ONE

Dear Editor, dear reviewers,

First of all, thank for your time and comments regarding our protocol. On behalf of the authors I would be delighted to answer all points raised from both the Editor and the Reviewer.

To start with, I have addressed the additional requirements pointed out by the journal in terms of 1) manuscript requirements, 2) data access, and 3) grant information. Amendments have been made accordingly. Furthermore, all funding statements have been removed from the manuscript.

Reviewers comments:

**1. Was the database search performed between September the 2^nd^ and September the 20^th^? Is the schedule of the protocol updated?**

I most certainly agree on the fact that the paragraph containing information on our literature search is not sufficient. Therefore, following amendments have been made at the top, line 99 to 105:

“The literature search will be conducted in two steps. First, we will use the SBU to screen for eligible articles. It consists of systematic literature searches on articles published between 1980 and the 10^th^ of January 2014 [12]. Secondly, in collaboration with a librarian, we will perform the exact same systematic literature search as in the SBU carried out in the following databases: National Library of Medicine (PubMed), Excerpta Medica Database (EMBASE), PsycInfo, Cumulative Index to Nursing and Allied Health Literature (CINAHL), Cochrane Library, and Web of Science. Our literature search will include articles published from January the.”

**2. Why did the authors restrict the language of retrieved studies? How to quantify possible losses resulting from the methodological choice? Is the language exclusion criteria (German, Spanish, Portuguese,…) reasonable?**

We will restrict the language of retrieved articles due to language barriers and hereby, we will not be able to quantify losses of potential relevant articles due to this choice. I have added following sentence to line 126 to clarify:

“…due to language barriers;”

Again, thank you your consideration of our revised protocol.

On behalf of the authors,

Alexander Jahn
